# All-trans retinoic acid enhances cytotoxicity of CIK cells against human lung adenocarcinoma by upregulating MICA and IL-2 secretion

**DOI:** 10.1038/s41598-017-16745-z

**Published:** 2017-11-28

**Authors:** Xiao-yan Fan, Peng-yu Wang, Chao Zhang, Yu-long Zhang, Yun Fu, Cong Zhang, Qiao-xia Li, Jie-na Zhou, Bao-en Shan, Dong-wei He

**Affiliations:** 1 Department of Oncology, Hebei General Hospital, Shijiazhuang, Hebei, 050000 People’s Republic of China; 2 Department of Clinical Bio-Cell, 4th Hospital, Hebei Medical University, Shijiazhuang, Hebei, 050000 People’s Republic of China; 3 Research Center, 4th Hospital, Hebei Medical University, Shijiazhuang, Hebei, 050000 People’s Republic of China; 4 Department of Surgery, Number One Hospital of Shijiazhuang, Shijiazhuang, Hebei, 050000 People’s Republic of China

## Abstract

To determine the growth inhibition capability of all-*trans* retinoic acid (ATRA) with cytokine-induced killer cells (CIKs), we evaluated their effects, alone and in combination, on human lung carcinoma A549 cells. CIKs treated with ATRA significantly inhibited cell growth. Additionally, CIK with ATRA synergistically inhibited migration and invasiveness, colony formation of A549 and NCI-H520 cells. Furthermore, analysis of apoptosis markers Bcl-2, Bax, Survivin and cleaved Caspase-3 showed that *Bcl-2* and *Survivin* mRNA levels significantly decreased, and that *Bax* mRNA significantly increased, in the CIK + ATRA-treated cells, with corresponding effects on their respective proteins. The involved mechanisms may be associated with upregulated expression of MHC class I-Related Chain (MICA) and interleukin (IL)-2. These results suggest that administration of combined CIK and ATRA is a potentially novel treatment for lung carcinoma.

## Introduction

Lung cancer is one of the most common malignancies, and leads to substantial mortality worldwide^1^. In China, the 5-year overall survival rate of patients with lung carcinoma has remained at a low level for the past 30 years^2^. As first-line treatments—surgery, chemotherapy and radiotherapy—often have limited effects on metastatic, locally advanced, or recurrent disease^3^, new therapeutic approaches are needed. Adoptive immunotherapy has become a widely promising approach for solid tumours^4,5^. Since the 1980s, lymphokine-activated killer cells, tumour-infiltrating lymphocytes cells, natural killer (NK) cells and cytokine-induced killer (CIK) cells have been extensively employed in adoptive immunotherapy^6^. Among them, CIK cells are *ex vivo*-expanded T lymphocytes with a mixed T-NK phenotype, and endowed with wide non-MHC-restricted antitumour activity^7–9^. Several clinical trials have established the feasibility and safety of CIK cell transfusions^10–12^. CIK cell transfusion is an effective supplement to chemotherapy and radiotherapy for cancer treatment, and can evoke significant responses in patients with gastric and lung tumours^13–15^. The therapeutic effects of CIK in various tumours are encouraging, although their mechanisms are not yet clear^10,16–19^.

All-*trans* retinoic acid (ATRA), an acid derivative of vitamin A (retinol), has been widely used in differentiation induction therapy in acute myelogenous leukaemia^20–22^. Several studies have shown ATRA to inhibit cell migration, cell-cycle procession, invasiveness and proliferation, and promote apoptosis^23–25^; and also improve CD4- and CD8-mediated, tumour-specific immune response, by differentiating immature myeloid cells into mature dendritic cells, macrophages, and granulocytes^26^. ATRA can also upregulate expression of MHC class I homologs MICA and MICB to enhance NK cell activity^27^. Especially, ATRA-induced expression of MICA and MICB can enhance CIK cell cytotoxicity^28^. Moreover, Engedal *et al*. reported ATRA to extend lifespans of activated T cells by inhibiting spontaneous apoptosis^29^. All of these data indicate an important role for ATRA in immune response and a potentially synergistic effect with CIK cells in cancer immunotherapy.

In the present study, we investigated the synergistic effects of CIK and ATRA in human lung cancer cells, and preliminarily explored the underlying mechanism. Our findings imply a novel strategy for treating lung tumours.

## Materials and Methods

### Ethics statement

The experimental protocol was approved by the 4^th^ hospital, Hebei Medical University. The experimental methods were carried out in accordance with the approved guidelines and regulations. The human peripheral blood samples were provided by volunteer donors, and their informed consents were obtained and approved by 4^th^ hospital, Hebei Medical University. The animals used in this study were maintained in accordance with the care guidelines of laboratory animals of Hebei Medical University.

### Cell culture

The human lung epithelial carcinoma cell line A549 were obtained from the Cellular Biology Institute of the Shanghai Academy of Sciences (Shanghai, China). The NCI-H520 cell line was a gift from professor Zhongning Zhu (Hebei Medical University, Shijiazhuang, China). A549 cells were cultured in RPIM1640 (Gibco, Carlsbad, CA, USA) containing 10% fetal bovine serum (FBS; Sijiqing, Shanghai, China), 100 U/ml penicillin and 100 μg/ml phytomycin at 37 °C and 5% CO_2_ in a humidified atmosphere incubator. The NCI-H520 cell line was maintained in Dulbecco’s modified Eagle’s medium (DMEM; Gibco, Carlsbad, CA, USA), supplemented with 10% FBS, 100 U/ml penicillin, 100 U/ml streptomycin and 2 mM L-glutamine. The cells were maintained in an incubator with 5% CO_2_ at 37 °C.

### CIK culture and expansion

Human peripheral blood samples were obtained from volunteer donors. Fresh peripheral blood mononuclear cells (PBMCs) were isolated by density gradient centrifugation using cell separation media Lymphoprep (Tianjin Chuanye Biotechnology Co., Ltd. Tianjin, China). PBMCs were cultured in GT-T551 (Takara, Japan) containing with 10% FBS (Sigma, USA) in 1000 U/ml human interferon (IFN)-γ1b (Miltenyi Biotec, Auburn, USA) overnight. After 24 h in culture at 37 °C and 5% CO_2_, 50 ng/ml LEAF^TM^ purified anti-CD3 antibody (Biolegend, San Diego, USA) and 300 U/ml recombinant human interleukin (IL)-2 (Shandong Quangang Pharmaceutical Co. Ltd, Shandong, China) were added. Fresh medium with IL-2 was added as needed. Finally, the expanded bulk CIK cells were analyzed by flow cytometry.

### Cell proliferation assay

The effects of CIK and ATRA (Sigma Chemical Co., St Louis, MO, USA), alone or in combination on cell proliferation was determined by MTS (3-(4,5-dimethylthiazol-2-yl)-5-(3-carboxymethoxyphenyl)-2-(4-sulphophenyl)-2H-tetazolium) assay according to the manufacturer’s instructions (Promega, Madison, WI, USA). Briefly, cells were treated with CIK (E:T ratio: 5:1, 10:1, 20:1 and 40:1, respectively), ATRA (1 × 10^−7^, 1 × 10^−6^, 1 × 10^−5^ and 1 × 10^−4^ mM, respectively), and CIK + ATRA (effector:target (E:T) ratio of CIK: 20:1; ATRA: 1 × 10^−5^ mM), in each well of plates (Gibco, USA). After incubation at the indicated time points at 37 °C in a humidified incubator, MTS solution was added (20 μl/well) to the wells, which were incubated again for 3 h at 37 °C. Finally, the absorbance at 492 nm was measured using a microplate reader (TitertekMultiskan, North Ryde, Australia) to determine the effect of CIK and ATRA, alone and in combination on cell viability. The inhibition rate (IR%) was calculated by the following equation: IR% = (A_control_ − A_experimental_)/A_control_ × 100%.

### Colony formation assay

To investigate the effects of CIK and ATRA, alone or in combination on the colony formation of A549 and NCI-H520 cells, the colony formation assay was performed. Briefly, about 600 cells were seeded on 60-mm dishes and cultured in a humidified incubator with 5% CO_2_ for 10 days at 37 °C. Then, the cells were wased with PBS 3 times and finally the cells were fixed with 4% paraformaldehyde for 20 min, and stained with 2% crystal violet for 15 min and counted under an inverted microscope. The assay was carried out in triplicate.

### *In vitro* cell migration and invasion assays

A wound-healing assay was performed to determine the cell migration rates of all groups. The cells were plated into 24-well plates and incubated to reach a final confluence of 100%. Then, wounds were carefully created using a sterile micropipette tip, and the wells were rinsed with serum-free medium three times. cells were treated with CIK (E:T ratio: 20:1), ATRA (1 × 10^−5^ mM), alone and in combination for 48 h. Finally, images were taken at 0 h and again after 48 h of treatment and the wound areas were measured.

Cell invasion analysis was also evaluated in a 24-well plate Transwell chamber (Corning Incorporated, USA). The Transwell was coated with 100 μl Matrigel and incubated at 37 °C for 1 h. Then, cells were treated with CIK and ATRA, alone or in combination for 48 h. Subsequently, the cells were trypsinized and resuspened in serum-free medium and seeded on the upper chamber of the Transwell, while 100 μl medium with 10% FBS was placed in the lower chambers. After incubation for 16 h in a 5% CO_2_ humidifed incubator at 37 °C, the Matrigel glue on the upper chamber was removed using a cotton swab. Next, the cells on the lower chamber were fixed in −20 °C methanol for 15 min, and stained with 1% crystal violet in PBS for 1 h at room temperature. The cells on the lower chamber were calculated as invasion cells in 5 random fields of each group.

### Flow cytometry for cell apoptosis

The effects of CIK + ATRA were assayed with an annexin V-phycoerythrin/7-amino-actinomycin D apoptosis detection kit (BD, San Jose, USA). Briefly, 1 × 10^6^ cells per sample were harvested and wased with PBS. Then, the samples were incubated with 5 μl annexin V-phycoerythrin and 5 μl 7-amino-actinomycin D for 15 min. Finally, the cells were analyzed with flow cytometry. The data were expressed as mean ± SD from three independent experiments.

### Real-time PCR assay

Assessment of mRNA expression in each group was performed as follows. Total cellular RNA was isolated using a TRI reagent RNA isolation reagent according to the manufacturer’s instructions (Sigma-Aldrich, St. Louis, MO, USA). A reverse transcription system (Promega) was used to generate first-strand template cDMA from 5 μg of total RNA. The PCR reaction was determined as follows: denaturation at 95 °C for 10 min, followed by 40 cycles of 95 °C for 15 s, 55 °C for 15 s, and 72 °C for 30 s. SYBR-Green qPCR Master Mix was used according to the manufacturer’s instructions (Thermo Fish Scientific, USA). The sequences of primers were as follows. B-cell lymphoma 2 (*Bcl-2*), 5′-CTGAAGACCCTCAGGATGCG-3′(sense) and 5′-TGGCCTTGTAGACACCTTGG-3′(antisense); *Bcl-2* associated X protein (*BAX*), 5′-GCTGAACCAAGGAGACGGAA-3′ (sense) and 5′-AGAAGTTGGCATGGTAGCCC-3′ (antisense); *Survivin*, 5′-GCTGAACCAAGGAGACGGAA-3′ (sense) and 5′-AGAAGTTGGCATGGTAGCCC-3′ (antisense); and GAPDH, 50-GTCAACGGATTTGGTCGTATTG-30 (sense) and 50-CATGGGTGGAATCATATTGGAA-30 (antisense). The expression of GAPDH was considered as a reference gene and as a cDNA integrity control.

### Western blot analysis

Cells were harvested in ice-cold RIPA butter and homogenized by sonication. After centrifugation, the protein lysates was routinely separated by electrophoresis and subsequently transferred into a PVDF membrance (Millipore, Germany). Then, after 1 h incubation in 10% nonfat milk in TBST (50 mM NaCl, 30 mM Tris-HCl, 0.1% Tween 20), the membranes were incubated with the primary antibody at 4 °C overnight (*Bcl-2*, Bax, cleaved-Capspase 3 and *Survivin*:1:1000, Cell Signaling, USA; GAPDH:1:10000, Santa Cruz, USA). Next, the membranes were incubated with the respective HRP-conjugated secondary antibodies (1:5000, Santa Cruz, USA). Finally, the membranes were developed with ECL Western Blotting Detection reagents to visualize chemifluorescense signals.

### *In vivo* tumour growth assay

Balb-c/null mice (4-week old, purchased from Beijing Weitonglihua Experimental Animal Co. Beijing, China) were maintained in a specific pathogen-free animal laboratory with a 12–12 h light/dark cycle (light on 07:00–19:00). Human A549 cells, about 5 × 10^6^ in 0.2 ml PBS, were s.c. injected into the right-side flank area of each mouse. When the tumours reached about 0.1 cm^3^, mice were randomly divided into 5 groups: (1) control group (0.9% NS, 10 ml/kg), (2) cis-platinum (CDDP) group (2 mg/kg), (3) CIK group (1.0 × 10^7^), (4) ATRA group (5 μmol/kg) (5) CIK + ATRA group. Each group contained 4 mice, and there was no difference in tumor size between the groups. Using this animal model, each mouse was given by intraperitoneal injection every 2 days. Tumour volumes were monitored once a week by using a digital caliper, and the tumour volume was recorded using the following formula: tumour volume (cm^3^) = (a × b^2^)/2, where a is the length and b is the width of the tumour. The therapy continued for 5 weeks and finally animals were kindly sacrificed and the tumours were removed and weighted.

### Flow cytometry assay

The expressions of MICA and MICB were determined by flow cytometry assay as follows. Cells were divided into 4 groups: (1) control group; (2) CIK group (E:T ratio: 20:1), (3) ATRA group (1 × 10^−5^ mM) (4) CIK + ATRA group. After treatment for 48 h, the cells in each group were harvested and incubated with either a phycoerythrin (PE)-labeled mouse anti-human MICA mAb (clone number 159227, R&D systems, USA) and a allophycocyanin (APC)-labeled mouse anti-human MICB mAb (clone number 236511, R&D systems, USA) on ice for 30 min. As isotype controls, cells were incubated with PE- or APC- labeled mouse IgG_2b_ antibodies. Finally, the cells were washed with FACS buffer and analyzed using a FACS Calibur flow cytometer (Becton Dickinson, CA, USA).

Additionally, the levels of soluble MICA in culture medium of each group were determined using DuoSet ELISA Development kits for MICA (R&D Systems, USA).

### ELISA assay

To investigate the content of IL-2 in the supernatants of A549 and NCI-H520 lung cancer cells and serum of balb-c/null mice treated with CIK (E:T ratio: 20:1) and ATRA (1 × 10^−5^ mM), alone or in combination, ELISA assay was performed according to the manufacturer’s instructions. Briefly, about 2 × 10^5^ cells treated with CIK and ATRA, alone or in combination, were seeded in 6-well plates. The plates were incubated in a 5% humidified incubator at 37 °C. After 24, 48 and 72 h, the supernatants were collected to detect the concentrations of IL-2.

### Statistical analysis

The data are reported as mean ± SD, and statistical significances between groups were performed by one-way analysis of variance (ANOVA). And Turkey’s method was used for multiple-group comparisons. All the experimental data were analyzed by SPSS 13.0 Software. A *p* value of less than 0.05 was considered as statistical significance.

## Results

### Phenotypic characterization of CIK cells

**Figure 1:**
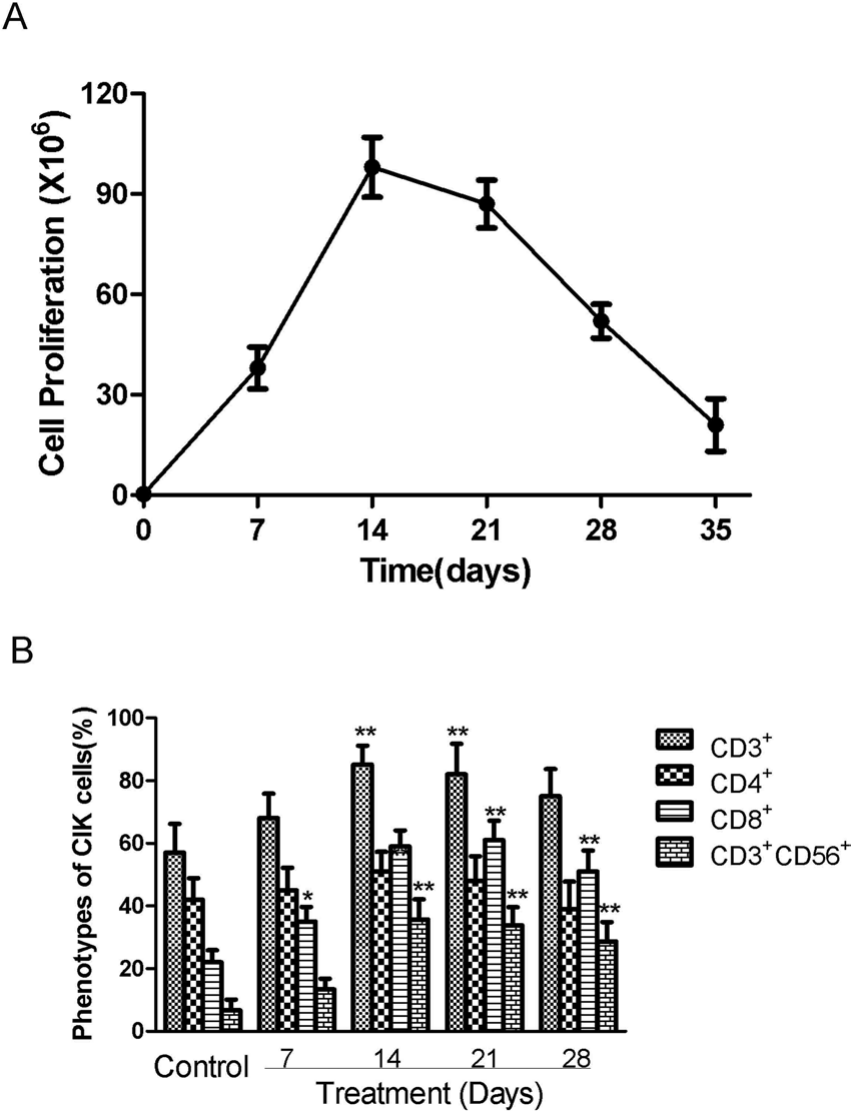
Characterization of CIK cells. (**A**) Cell proliferation curve of CIK cells treated by CD3, IFN-γ1b and IL-2. (**B**) Cell phenotypes of CIK cells. The data presented are mean ± SD from three independent experiments. *p < 0.05,**p < 0.01 compared with control groups, respectively.

PBMCs were cultured for about 3 weeks with the timed additions of IFN-γ1b, anti-CD3 and IL2. At the end of the expansion, immunophenotypic CIK cells were analysed (Fig. 1). Although the main fraction was CD3^+^ cells (85.1 ± 6.1%), the antitumour CIK cells were the main CD3^+^/CD56^+^ fraction (35.7 ± 5.7% on Day 14 and 28.7 ± 6.2% on Day 21 (Fig. 1B).

### Effects of CIK and ATRA, alone or in combination on viability of viability of A549 and NCI-H520 cells

**Figure 2:**
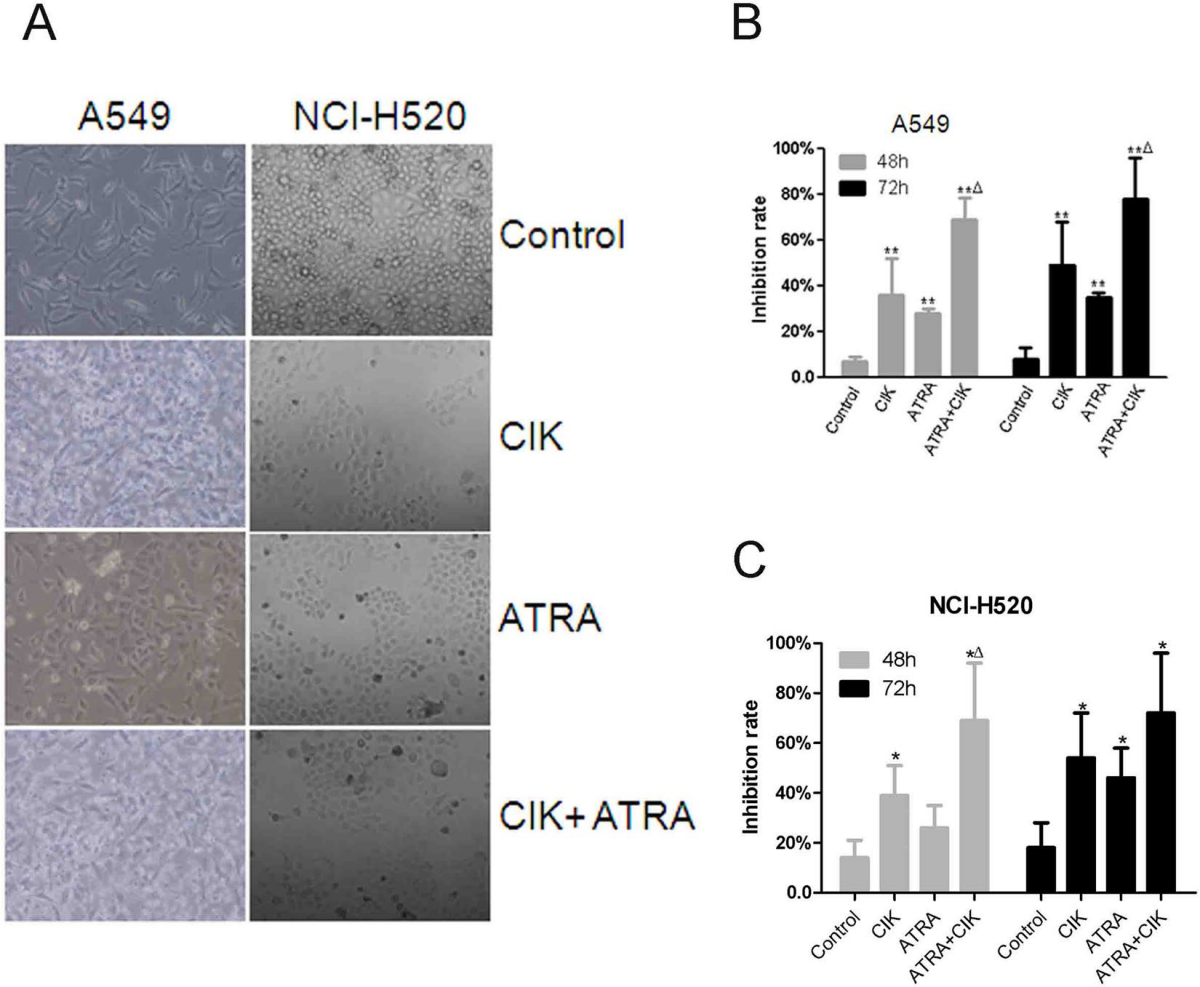
Synergistic effects of CIK and ATRA in human A549 and NCI-H520 lung carcinoma cells. (**A**) Morphological changes of A549 and NCI-H520 cells treated with CIK and ATRA, alone or in combination. (**B**,**C**) Cell inhibition rates of A549 and NCI-H520 cells induced by CIK or ATRA, or in combination, respecitively. The data presented are mean ± SD from at least three independent experiments. Compared with control group, significant difference in cell prolifiertion inhibition was found among these cells (p < 0.01). *p < 0.05,**p < 0.01 compared with control groups, respectively. ^Δ^p < 0.05 compared with CIK or ATRA group, respectively.

To evaluate the synergistic effects of CIK combined with ATRA on cell viability, A549 and NCI-H520 cells were treated with CIK and ATRA, alone or in combination, using MTS assay after 24, 48 and 72 h. Morphological changes in A549 and NCI-H520 cells treated with CIK and ATRA, alone or in combination were shown in Fig. 2A. CIK cells alone markedly inhibited the growth of A549 and NCI-H520 cells in an effector:target (E:T) ratio- and time-dependent manner (Fig. 2B,C). Similarly, ATRA also inhibited the cell proliferation in a dose- and time- dependent manner. As expected, treatment of CIK and ATRA in combination significantly inhibited cell growth after 48 or 72 h (Fig. 2B,C).Furthermore, the synergistic effects of CIK and ATRA against A549 or NCI-H520 cells were markedly higher than those of CIK or ATRA groups, respectively.

### Effects of CIK combined with ATRA on cell migration and invasion of A549 and NCI-H520 cells

**Figure 3:**
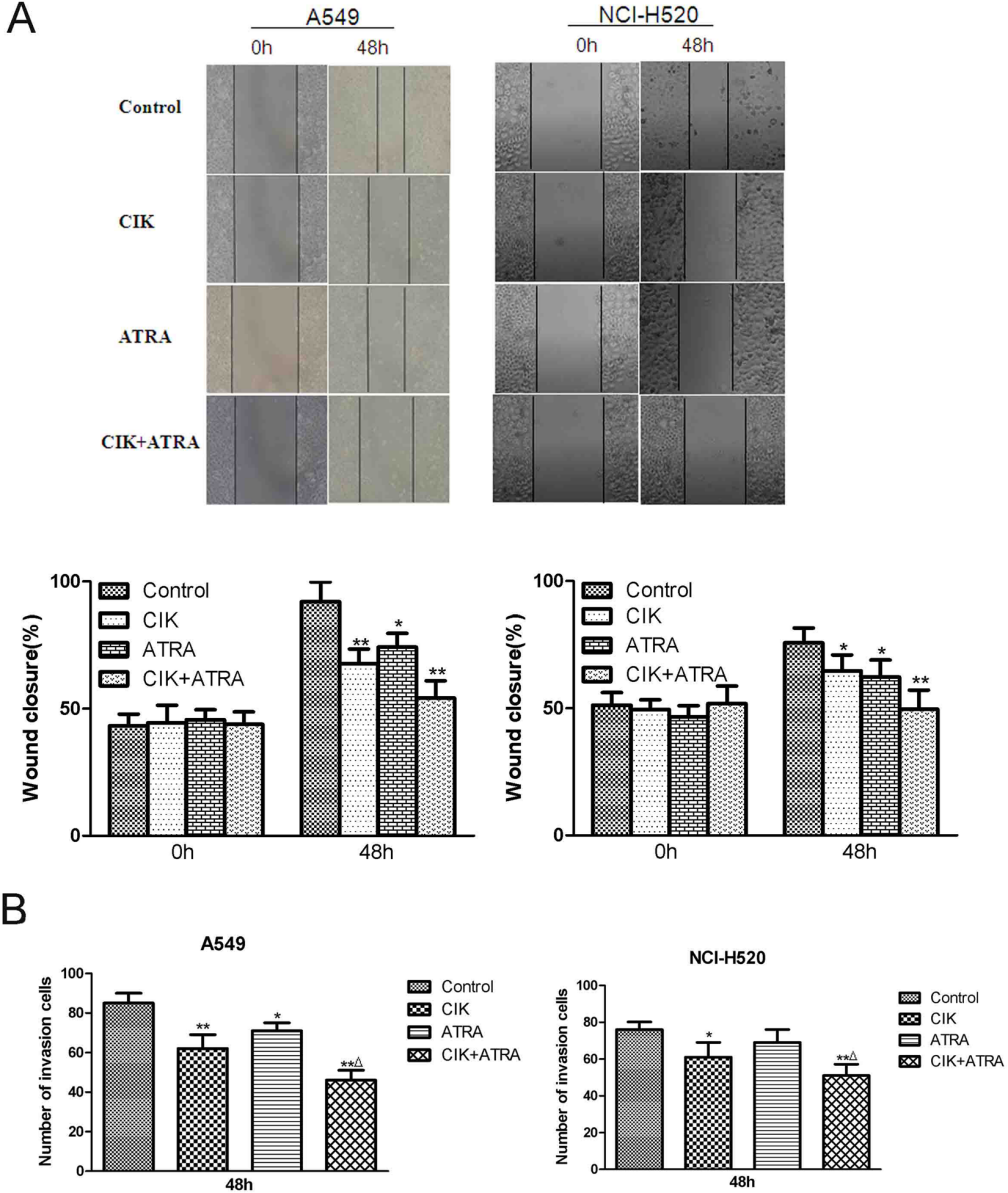
Effects of CIK combined with ATRA on cell migration (**A**) and invasion (**B**) of A549 and NCI-H520 cells. The wound closure percentage was measured using Axio Vison software. The cells on the lower chamber were calculated as invasion cells in 5 random fields of each group. The experimental data presented are mean ± SD from at least 3 independent experiments.*p < 0.05,**p < 0.01 compared with control groups, respectively. ^Δ^p < 0.05 compared with CIK or ATRA group, respectively.

To investigate the effects of CIK and ATRA, alone or in combination on the mobility ability of cells after treatment for 48 h, wound healing assay was performed (Fig. 3A). The scratch assay analysis revealed that CIK and ATRA alone showed markedly effects on the cell migration of A549 and NCI-H520 cells (p < 0.05). Furthermore, CIK and ATRA in combination treatment showed a more significant influence on the cell migration of A549 and NCI-H520 cells (p < 0.01). Transwell invasion assays were also performed to observe the effect of CIK and ATRA induced cell invasion (Fig. 3B). After treatment for 48 h, the number of CIK and ATRA in combination treated A549 or NCI-H520 cells were significantly less than those of the controls (p < 0.01) or CIK, ATRA alone group (p < 0.05). Taken together, these data revealed that the migration and invasion of A549 and NCI-H520 cells were significantly inhibited after synergistic treatment with CIK combined with ATRA for 48 h.

### Effects of combined CIK + ATRA on colony formation and apoptosis of A549 and NCI-H520 cells

**Figure 4:**
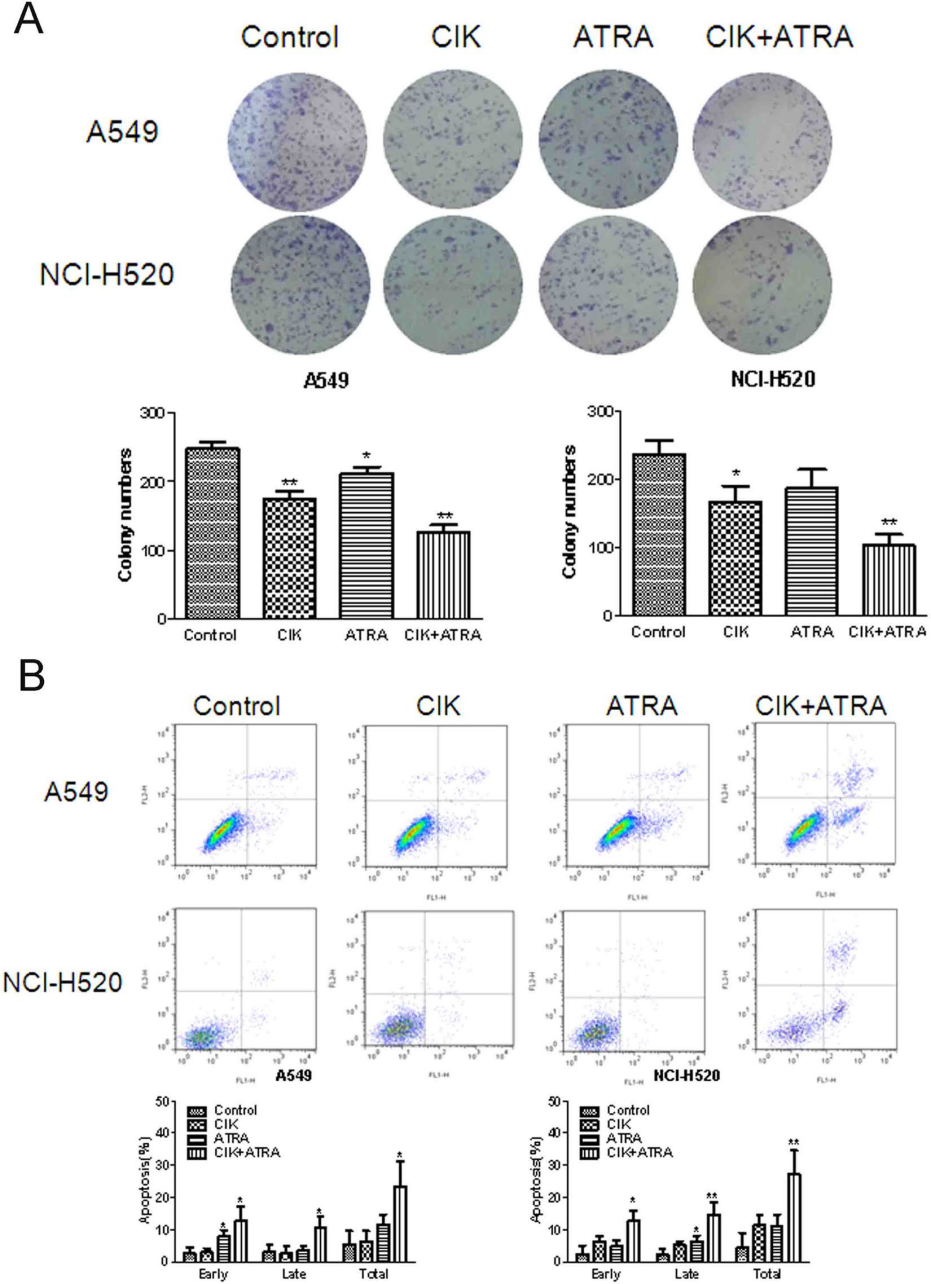
Effects of CIK combined with ATRA on colony formation and cell apoptosis of A549 and NCI-H520 cells. (**A**) Cell colony formation was significantly inhibited by treatment with CIK combined with ATRA. (**B**) CIK combined with ATRA treatment effectly increased the total apoptosis of A549 and NCI-H520 cells detected by annexin V-phycoerythrin/7-amino-actinomycin D flow cytometry assay. CIK or ATRA alone treatment also increased the apoptosis to a certain extent at the early or late stage. The number of colonies was calculated and plotted on the histogram (n = 3). *p < 0.05,**p < 0.01 compared with control groups, respectively. ^Δ^p < 0.05 compared with CIK or ATRA group, respectively.

After treatment with combined CIK + ATRA, numbers and volumes of colonies of A549 and NCI-H520 cells were significantly reduced (*P* < 0.01; Fig. 4A). These reductions were more pronounced than in cells treated with CIK or ATRA alone (*P* < 0.05 for both).

Apoptosis has an important function in controlling cancer. The synergistic effects of combined CIK + ATRA were significantly higher than those seen in controls, or in cells treated with CIK or ATRA alone (Fig. 4B).

### Effects of combined CIK + ATRA on bcl-2, Bax and survivin mRNA levels

**Figure 5:**
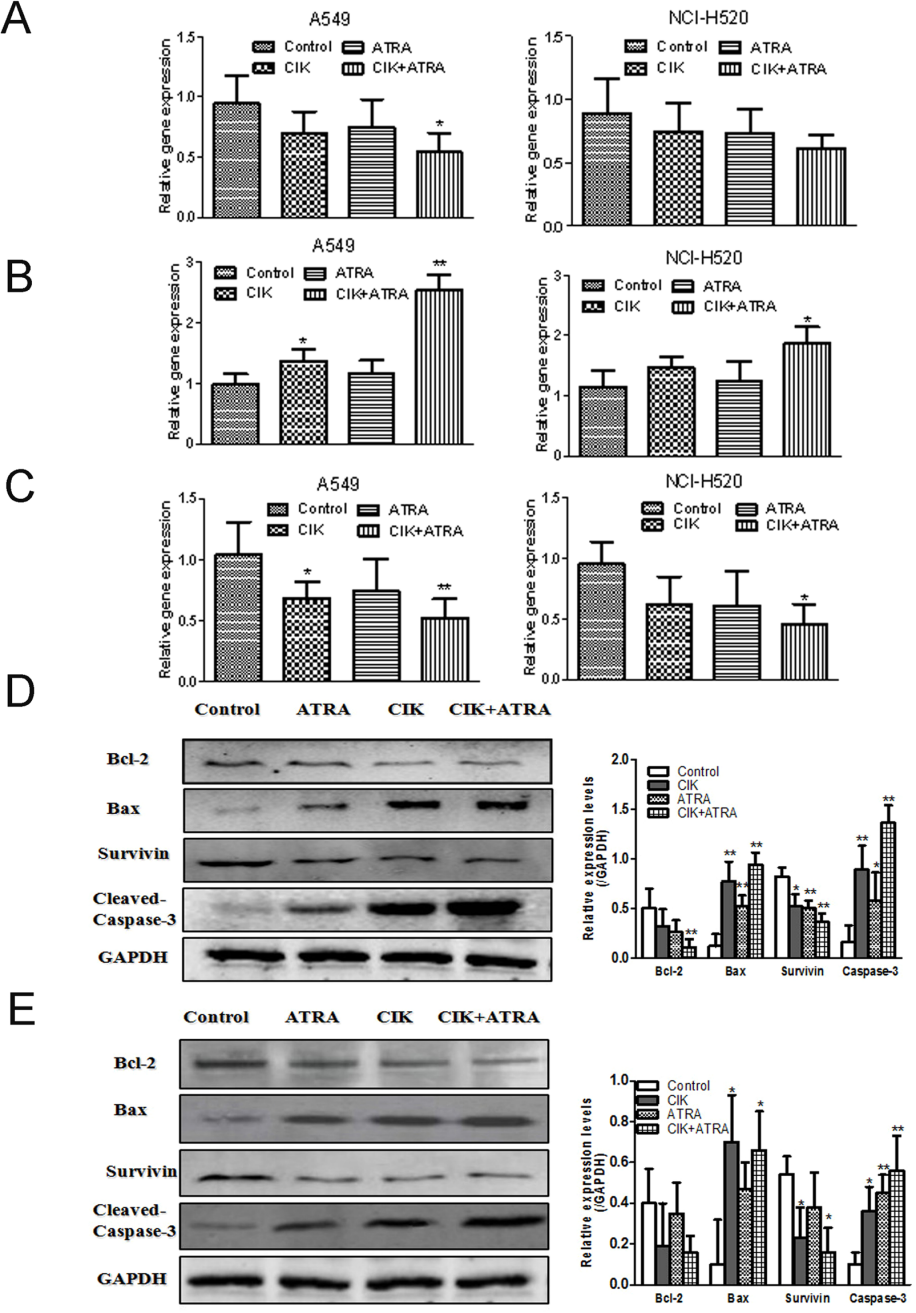
Effects of CIK combined with ATRA on the (**A**) *Bcl*-2, (**B**) *Bax* and (**C**) *Survivin* expression at mRNA levels (**A**–**C**) and protein levels (**D**,**E**) of A549 and NCI-H520 cells. Quantitative RT-PCR and Western blot assays were used to determined the changes of mRNA and protein levels. The data presented are means ± SD from at least 3 independent experiments. GAPDH was utilized for an endogenous reference to standardize mRNA or protein expression levels. *p < 0.05,**p < 0.01 compared with the control group, respectively.

The Bcl-2 family mediates apoptosis-related mitochondrial events. Using real-time quantitative reverse transcriptase-polymerase chain reaction, we determined the mRNA levels of *Bcl-2*, *Bax* and *Survivin* in A549 and NCI-H520 cells (Fig. 5A–C). Expression levels were standardized against those of glyceraldehyde-3-phosphate dehydrogenase (*GAPDH*). After treatment with CIK or ATRA for 48 h, mRNA levels of *Bcl-2*, and *Survivin* in A549 or NCI-H520 cells slightly decreased, and levels of *Bax* increased, compared with controls. Furthermore, after treatment with combined CIK + ATRA, significant differences were observed in mRNA levels of *Bcl-2* (*P* < 0.05), *Bax* (*P* < 0.01) and *Survivin* (*P* < 0.01). The results suggested that combined CIK + ATRA could downregulate mRNA of apoptosis-inhibiting *Bcl-2* and *Survivin*, and upregulate apoptosis-promoting *Bax* mRNA.

### Effects of combined CIK + ATRA on Bcl-2, Bax, Survivin and Caspase-3 protein

To further evaluate the mechanism by which CIK and ATRA affect cell apoptosis, changes in protein expression of apoptosis-related proteins, including Bcl-2, Bax, Survivin and cleaved Caspase-3, were examined by western blot assays. When treated with CIK or ATRA alone, levels of Bcl-2 were not significantly changed (*P* > 0.05), levels of Survivin were significantly decreased (*P* < 0.05), and levels of Bax and cleaved Caspase-3 were significantly increased (*P* < 0.05), compared to controls (Fig. 5D,E). Furthermore, when treated with combined CIK + ATRA, protein levels of Bax and Casepase-3 were significantly upregulated (*P* < 0.05), and levels of Bcl-2 and Survivin were significantly downregulated (*P* < 0.05).

### Effects of combined CIK + ATRA on tumour growth *in vivo*

**Figure 6:**
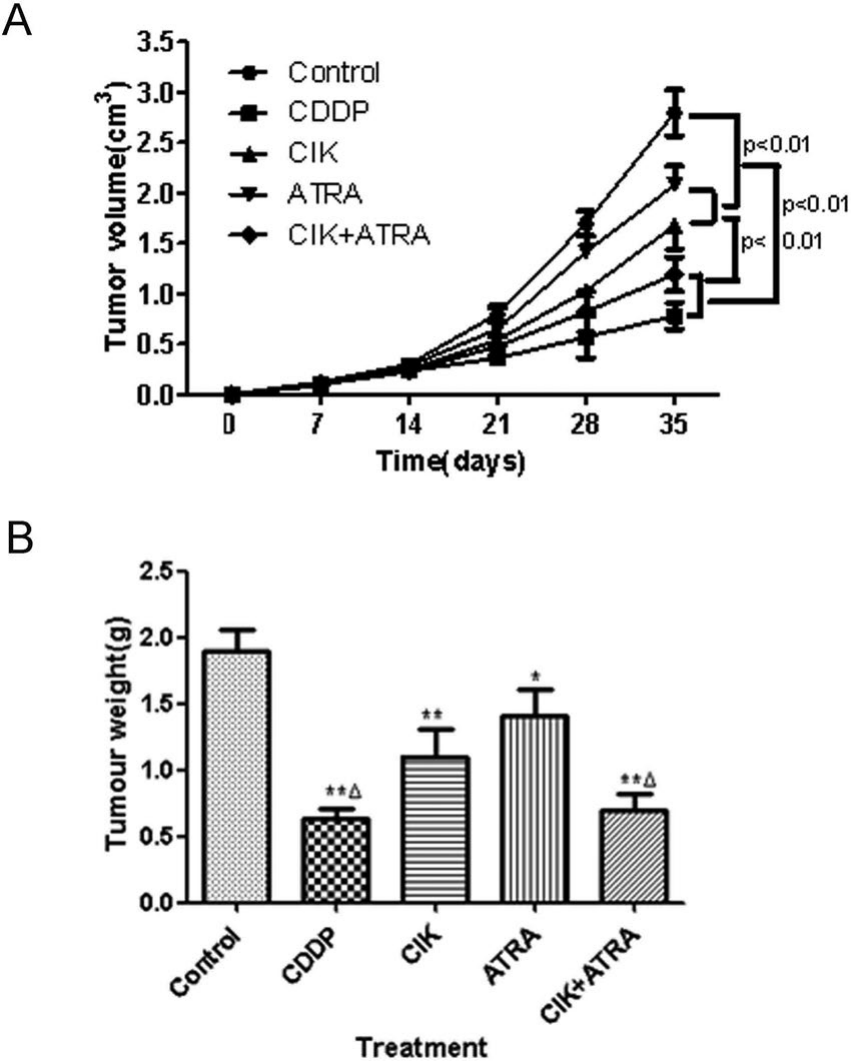
CIK combined with ATRA treatment inhibited tumor growth of A549 cells *in vivo*. (**A**) Tumor Volume curve and (**B**) Tumor weight graph. 20 mice were randomly divided into 5 groups: (1) control group (0.9% NS, 10 ml/kg), (2) cis-platinum (CDDP) group (2 mg/kg), (3) CIK group (1.0 × 107), (4) ATRA group (5 μmol/kg). (5) CIK + ATRA group. Human A549 cells, about 5 × 10^6^ in 0.2 ml PBS, were s.c. injected into the right-side flank area of each mouse. After treatment, tumour volumes and weights of mice in each group were determined. *p < 0.05,**p < 0.01 compared with the control group, respectively. ^Δ^ p < 0.01 compared with CIK or ATRA group.

In this study, nude mice were used to assess effects of CIK and ATRA, alone or in combination on tumour growth *in vivo*. In A549 xenograft-bearing nude mice, tumour volume growth in each treated group was significantly inhibited in a time-dependent manner compared with controls (Fig. 6A). The CIK-alone and ATRA-alone groups had significantly decreased tumour volumes by Day 35 (*P* < 0.01); and the combined CIK + ATRA had significantly lower tumour volume, compared with CIK-only and ATRA-only groups (*P* < 0.01). Additionally, median tumour weights in mice treated with CDDP, CIK and ATRA, alone or in combination, were significantly lower than those in the control group (Fig. 6B). Compared with the CIK-only and ATRA-only groups, median tumour weights in the combined CIK + ATRA group were significantly less (*P* < 0.01).

### Effects of MICA and MICB surface ligands and soluble forms in cells treated with CIK and ATRA, alone or in combination

**Figure 7:**
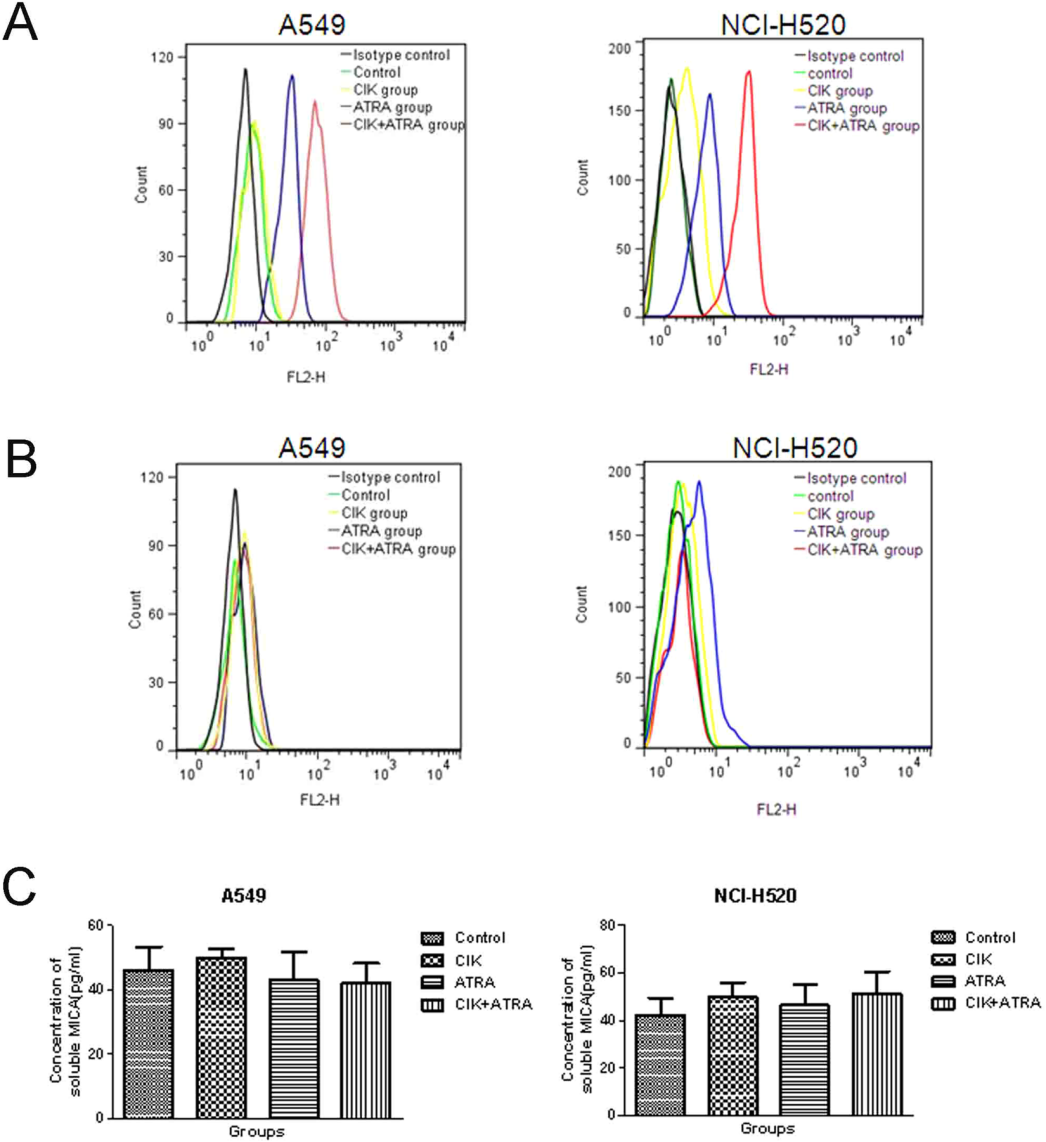
Effects of CIK and ATRA, alone or in combination, on the expression of MICA and MICB. Representative flow cytometric profiles of cell surface MICA (**A**) and MICB (**B**). The black profile indicates a control profile of A549 or NCI-H520 cells incubated with mouse IgG_2b_. (**C**) Effects of CIK and ATRA, alone or in combination, on the secretion of soluble MICA. Cells were treated for 48 h and the concentrations of soluble MICA in the medium were determined. The data presented are mean ± SD from at least three independent experiments.

Cells were treated with CIK and ATRA, alone or in combination for 48 hours, and expression of cell-surface ligands, MICA and MICB, and their soluble forms in media were determined (Fig. 7). MICA was highly expressed in cells treated with combined CIK + ATRA (*P* < 0.05), whereas MICB expression was very low (Fig. 7A,B). ATRA alone significantly increased expression of cell surface MICA (*P* < 0.05), but not MICB.

Amounts of soluble MICA were detected in the media of each group. Concentrations of soluble MICA in ATRA and CIK + ATRA groups changed slightly, but not significantly, in the media of all groups (*P* > 0.05; Fig. 7C).

### Greater IL-2 secretion in cells and nude mice treated with combined CIK + ATRA

**Figure 8:**
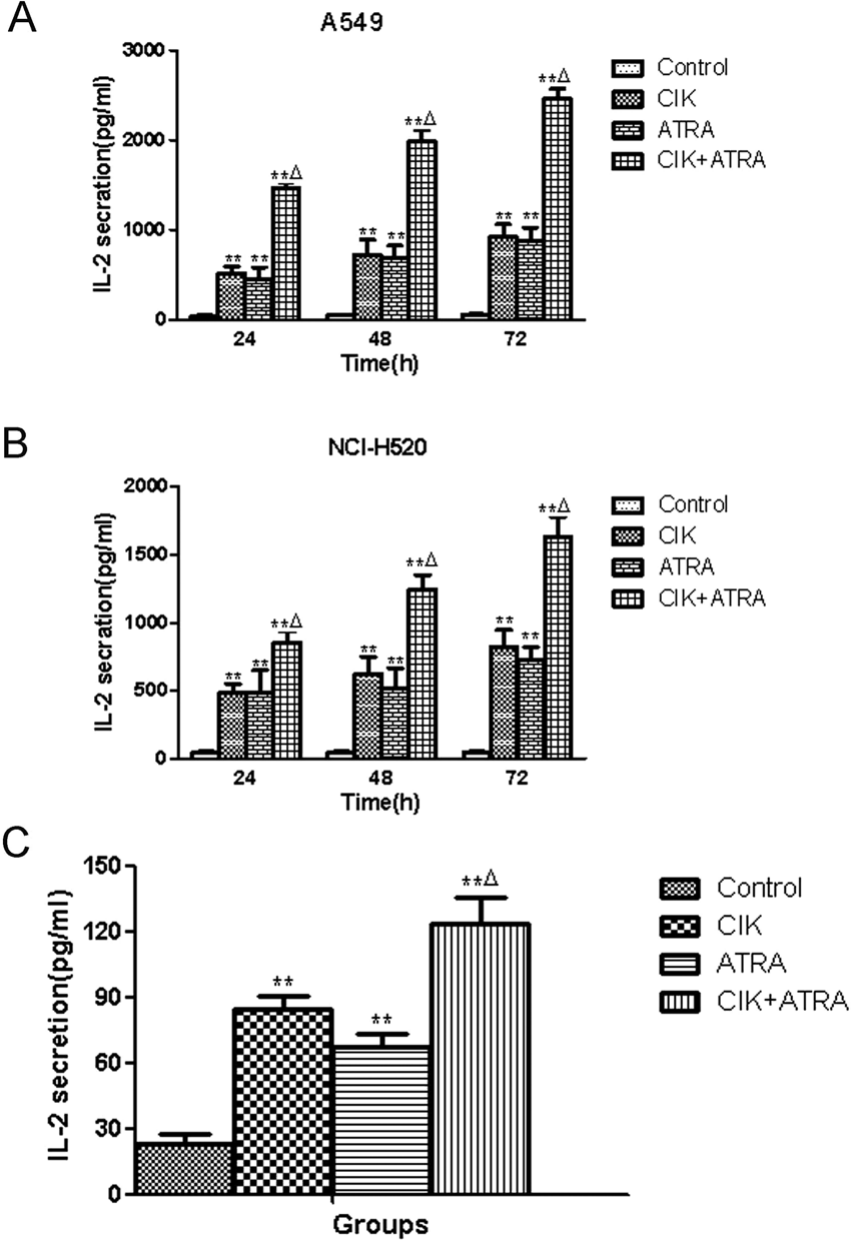
The content of IL-2 secretion in the cell supernatants of A549 and NCI-H520 cells (**A**,**B**) and nude mice (**B**) treated with CIK and ATRA, alone or in combination. The levels IL-2 in each group were determined by ELISA. Vertical bars indicated the mean ± SD of three independent experiments. *p < 0.05,**p < 0.01 compared with the control group, respectively. ^Δ^p < 0.01 compared with CIK or ATRA group, respectively.

To help clarify the mechanism of the combined CIK + ATRA synergistic effect on human lung cancer, IL-2 secretion in A549 and NCI-H520 cell and nude mice sera were determined by ELISA. Levels of IL-2 secretion in A549 and NCI-H520 cells (Fig. 8A,B) and nude mice (Fig. 8C) treated with combined CIK + ATRA were significantly higher than in other groups (*P* < 0.01), which implies that increased IL-2 secretion is involved in the synergistic effect of combined CIK + ATRA.

## Discussion

Previous studies have shown immunotherapy to be a promising treatment for tumours^10,19^, and should be an effective complement to chemotherapy or radiotherapy^5,30^. ATRA was initially used to treat acute promyelocytic leukaemia, and many studies have shown ATRA to cause significant tumour regression, although the underlying mechanism is unclear^24,31,32^. Some studies have shown ATRA to inhibit progression of breast cancer^33^. Moreover, ATRA can reverse epithelial–mesenchymal transition (EMT) of hepal-6 cells by regulating EMT marker genes^25^. ATRA also can inhibit RKO colorectal cancer cell migration by downregulating myosin light-chain kinase expression via the extracellular signal-regulated kinase-1/Mitogen-activated protein kinase signalling pathway^34^. Furthermore, ATRA may partially downregulate matrix metalloproteinase expression to induce glioma cell invasion^23^. However, exiguous data is available on the treatment of solid tumours with combined CIK + ATRA. In the present study, combined CIK + ATRA was used on lung carcinoma cells and *in vivo* tumours; our results clearly showed that ATRA could enhance the efficiency of CIK cells in killing human lung carcinomas.

CIK immunotherapy is a novel therapeutic approach, with the following advantages: (1) strong antitumour activity, (2) broad spectrum of tumour targets and (3) weak side-effects.

We found the E:T ratio of CIK cells to be pivotal in controlling cancer growth. Here, cells were treated with CIK cells at E:T ratios of 5:1, 10:1, 20:1 and 40:1, for 24, 48 and 72 hours. After assessing deaths of targeted cells, we selected CIK cells at E:T ratio: 20:1 for 48 hours as the suitable dose and time in combination with ATRA. Cancer cells were similarly treated with different concentrations of ATRA to obtain the suitable efficiency of ATRA in combination with CIK cells. Finally, CIK (E:T ratio: 20:1) and ATRA (1 × 10^−5^ mM) were used to investigate their synergistic efficiency on A549 and NCI-H520 cells. Cell colony formation was significantly inhibited by treatment with combined CIK + ATRA (Fig. 4A). Our data demonstrated that ATRA enhanced the killing ability of CIK cells for A549 and NCI-H520 lung carcinoma cells, though the related mechanism remains to be elucidated.

Metastasis is a hallmark of advanced cancer, and depends on cell migration and invasion to their surrounding vessels and tissues. The anti-migration and -invasion properties of ATRA have been widely shown in recent studies^23,25^. In the present study, the wound-healing assay showed that combined CIK + ATRA significantly inhibited migration of A549 lung cancer cells. ATRA could inhibit cell migration by reducing expression of myosin light-chain kinase^34^ or matrix metalloproteinase^23^, or by reversing EMT^25^. At the same time, CIK cells can directly kill metastatic cells that escape immune surveillance, thus significantly reducing cell migration.

Apoptosis is a form of programmed cell death that eliminates cells without releasing harmful substances into the surrounding environments. In cancer cells, apoptosis is often deregulated, leading to rapid proliferation and tumour growth, which limits the effects of conventional therapy^35,36^. Combined CIK + ATRA increased total apoptosis in A549 and NCI-H520 cells, as detected by annexin V-phycoerythrin/7-amino-actinomycin D flow cytometry assay (Fig. 4B). The Bcl-2 family of intracellular proteins regulate apoptosis by controlling mitochondrial membrane permeability^36^, and are considered to control caspase activation. Caspase-3 is the caspase most directly associated with apoptosis, but is activated by the initiator caspases, (caspase-8, caspase-9 and caspase-10)^36^. After CIK and ATRA treatment (alone and in combination), real-time PCR and western blot were used to detect variations in expressions of Bcl-2, Bax, Survivin and Caspase-3. Our results indicate that combined CIK + ATRA decreased expression of anti-apoptotic proteins (Bcl-2 and Survivin), increased pro-apoptotic proteins (Bax and cleaved Caspase-3), and significantly increased apoptosis of A549 and NIC-H520 cells.

In humans, NKG2D is expressed on most natural killer cells, γδ-T cells and CD8^+^/αβ-T cells^37^. Reportedly, NKG2D mediates both NK cell-induced cytotoxicity against target cells^38,39^, and CIK-cell cytotoxicity against multiple myleoma cells^28,40^. As ligands of NKG2D, the major histocompatibility complex class I homologues MICA and MICB function as signals of cellular stress^41^; upregulated MICA can directly improve CIK-cell cytotoxicity against multiple myeloma^28^. Interestingly, ATRA reportedly increases expression of MICA and MICB^27^, which implies that ATRA may enhance CIK cytotoxicity by upregulating MICA expression. Here, significantly high expression of MICA, but not MICB, was found in the combined CIK + ATRA group, which suggests that the increased cytotoxicity of CIK cells was mainly mediated by MICA. MICA has been widely reported to be secreted into culture media as soluble MICA in HeLa, CALO, U-937, THP-1 and multiple myeloma cancer cells^28,42,43^. Furthermore, soluble MICA could decrease responsiveness of effector T cells by endocytosis, through binding to NKG2D receptors^41^. In this study, as MICA was identified on surfaces of A549 and NCI-H520 cells, soluble MICA was also detected by ELISA. The ATRA-only and combined CIK + ATRA groups did not significantly differ in soluble MICA levels, indicating that ATRA does not inhibit MICA shedding in A549 or NCI-H520 cells. Additionally, in contrast to another report^27^, ATRA alone did not increase MICB expression. The contradictory results are likely associated with differences in ATRA doses, cell lines or tumour type.

IL-2 has important functions in the tumour microenvironment, and ATRA may affect its production. Ertesvag *et al*. established that ATRA could stimulate human T-cell proliferation by increasing IL-2 secretion through mechanisms involving retinoic acid receptors^43^. Additionally, Engedal *et al*. reported that ATRA-induced IL-2 could extend the lifespan of activated T cells by inhibiting spontaneous apoptosis^29^. CIK cells stimulated with a combination of IL-2 and IL-15 have shown greater cytotoxicity against human lung cancer cells^3^. These results suggest that ATRA enhances the cytotoxic capacity of CIK by promoting IL-2 secretion. In the present study, levels of IL-2, *in vitro* and *in vivo*, were measured in each group; highly elevated levels of IL-2 were found in the combined CIK + ATRA groups.

In conclusion, our data provide the first evidence that combining CIK with ATRA strongly and synergistically affects cell proliferation, migration and apoptosis of A549 and NCI-H520 lung cancer cells, both *in vitro* and *in vivo*. The involved mechanisms may be associated with upregulated MICA and IL-2 secretion. Therefore, combined CIK + ATRA could form a novel treatment for human lung carcinoma.

## Electronic supplementary material

Supplementary Information

## References

[1] Siegel RL, Miller KD, Jemal A (2016). Cancer statistics, 2016. CA Cancer J Clin.

[2] Jemal A, Siegel R, Xu J, Ward E (2010). Cancer statistics, 2010. CA Cancer J Clin.

[3] Wei C (2014). The CIK cells stimulated with combination of IL-2 and IL-15 provide an improved cytotoxic capacity against human lung adenocarcinoma. Tumour Biol.

[4] Stroncek D (2010). Developments in clinical cell therapy. Cytotherapy.

[5] Wang X (2014). Can the dual-functional capability of CIK cells be used to improve antitumor effects?. Cell Immunol.

[6] Ruella M, Kalos M (2014). Adoptive immunotherapy for cancer. Immunol Rev.

[7] Schmidt-Wolf IG, Negrin RS, Kiem HP, Blume KG, Weissman IL (1991). Use of a SCID mouse/human lymphoma model to evaluate cytokine-induced killer cells with potent antitumor cell activity. J Exp Med.

[8] Rosenberg SA (1985). Observations on the systemic administration of autologous lymphokine-activated killer cells and recombinant interleukin-2 to patients with metastatic cancer. N Engl J Med.

[9] Schmidt RE, Murray C, Daley JF, Schlossman SF, Ritz J (1986). A subset of natural killer cells in peripheral blood displays a mature T cell phenotype. J Exp Med.

[10] Hontscha C, Borck Y, Zhou H, Messmer D, Schmidt-Wolf IG (2011). Clinical trials on CIK cells: first report of the international registry on CIK cells (IRCC). J Cancer Res Clin Oncol.

[11] Sangiolo D (2011). Cytokine induced killer cells as promising immunotherapy for solid tumors. J Cancer.

[12] Hui KM (2012). CIK cells–current status, clinical perspectives and future prospects–the good news. Expert Opin Biol Ther.

[13] Niu Q (2011). Cord blood-derived cytokine-induced killer cells biotherapy combined with second-line chemotherapy in the treatment of advanced solid malignancies. Int Immunopharmacol.

[14] Shi L (2012). Efficacy of adjuvant immunotherapy with cytokine-induced killer cells in patients with locally advanced gastric cancer. Cancer Immunol Immunother.

[15] Wu C, Jiang J, Shi L, Xu N (2008). Prospective study of chemotherapy in combination with cytokine-induced killer cells in patients suffering from advanced non-small cell lung cancer. Anticancer Res.

[16] Mesiano G (2012). Cytokine-induced killer (CIK) cells as feasible and effective adoptive immunotherapy for the treatment of solid tumors. Expert Opin Biol Ther.

[17] Olioso P (2009). Immunotherapy with cytokine induced killer cells in solid and hematopoietic tumours: a pilot clinical trial. Hematol Oncol.

[18] Schmidt-Wolf IG (1999). Phase I clinical study applying autologous immunological effector cells transfected with the interleukin-2 gene in patients with metastatic renal cancer, colorectal cancer and lymphoma. Br J Cancer.

[19] Dougan M, Dranoff G (2009). Immune therapy for cancer. Annu Rev Immunol.

[20] Castaigne S (1990). All-trans retinoic acid as a differentiation therapy for acute promyelocytic leukemia. I. Clinical results. Blood.

[21] Warrell RP (1991). Differentiation therapy of acute promyelocytic leukemia with tretinoin (all-trans-retinoic acid). N Engl J Med.

[22] Breitman TR, Collins SJ, Keene BR (1981). Terminal differentiation of human promyelocytic leukemic cells in primary culture in response to retinoic acid. Blood.

[23] Liang C, Yang L, Guo S (2015). All-trans retinoic acid inhibits migration, invasion and proliferation, and promotes apoptosis in glioma cells *in vitro*. Oncol Lett.

[24] Hsu SL, Hsu JW, Liu MC, Chen LY, Chang CD (2000). Retinoic acid-mediated G1 arrest is associated with induction ofp27 (Kip1) and inhibition of cyclin-dependent kinase 3 in human lung squamous carcinoma CH27 cells. Exp Cell Res.

[25] Cui J (2016). All-trans retinoic acid inhibits proliferation, migration, invasion and induces differentiation of hepa1-6 cells through reversing EMT *in vitro*. Int J Oncol.

[26] Kusmartsev S (2003). All-trans-retinoic acid eliminates immature myeloid cells from tumor-bearing mice and improves the effect of vaccination. Cancer Res.

[27] Jinushi M (2003). Expression and role of MICA and MICB in human hepatocellular carcinomas and their regulation by retinoic acid. Int J Cancer.

[28] Nwangwu, C. A., Weiher, H. & Schmidt-Wolf, I. G. Increase of CIK cell efficacy by upregulating cell surface MICA and inhibition of NKG2D ligand shedding in multiple myeloma. *Hematol Oncol*. 1–7 (2016). 10.1002/hon.2326 27430430

[29] Engedal N, Ertesvag A, Blomhoff HK (2004). Survival of activated human T lymphocytes is promoted by retinoic acid via induction of IL-2. Int Immunol.

[30] Li W (2013). Cytokine-induced killer cell therapy for advanced pancreatic adenocarcinoma: A case report and review of the literature. Oncol Lett.

[31] Huang ME (1988). Use of all-trans retinoic acid in the treatment of acute promyelocytic leukemia. Blood.

[32] Siddikuzzaman Guruvayoorappan C, Berlin Grace VM (2011). All trans retinoic acid and cancer. Immunopharmacol Immunotoxicol.

[33] Zuo L (2016). All-Trans Retinoic Acid Inhibits Human Colorectal Cancer Cells RKO Migration via Downregulating Myosin Light Chain Kinase Expression through MAPK Signaling Pathway. Nutr Cancer.

[34] Hanahan D, Weinberg RA (2000). The hallmarks of cancer. Cell.

[35] Cory S, Adams JM (2002). The Bcl2 family: regulators of the cellular life-or-death switch. Nat Rev Cancer.

[36] Bauer S (1999). Activation of NK cells and T cells by NKG2D, a receptor for stress-inducible MICA. Science.

[37] Carbone E (2005). HLA class I, NKG2D, and natural cytotoxicity receptors regulate multiple myeloma cell recognition by natural killer cells. Blood.

[38] Zafirova B, Wensveen FM, Gulin M, Polic B (2011). Regulation of immune cell function and differentiation by the NKG2D receptor. Cell Mol Life Sci.

[39] Lu X (2012). Role of NKG2D in cytokine-induced killer cells against multiple myeloma cells. Cancer Biol Ther.

[40] Groh V, Wu J, Yee C, Spies T (2002). Tumour-derived soluble MIC ligands impair expression of NKG2D and T-cell activation. Nature.

[41] Huang B, Sikorski R, Sampath P, Thorne SH (2011). Modulation of NKG2D-ligand cell surface expression enhances immune cell therapy of cancer. J Immunother.

[42] Weiss-Steider B, Soto-Cruz I, Martinez-Campos CA, Mendoza-Rincon JF (2011). Expression of MICA, MICB and NKG2D in human leukemic myelomonocytic and cervical cancer cells. J Exp Clin Cancer Res.

[43] Ertesvag A, Engedal N, Naderi S, Blomhoff HK (2002). Retinoic acid stimulates the cell cycle machinery in normal T cells: involvement of retinoic acid receptor-mediated IL-2 secretion. J Immunol.

