# Supplementary material for: All-trans retinoic acid enhances cytotoxicity of CIK cells against human lung adenocarcinoma by upregulating MICA and IL-2 secretion
Source: Sci Rep. 2017 Nov 28;7:16481. doi: 10.1038/s41598-017-16745-z (PMC5705634; doi:10.1038/s41598-017-16745-z)
Supplement: Supplementary file 1 — Supplementary Information [file 41598_2017_16745_MOESM1_ESM.pdf]

All-trans retinoic acid enhances cytotoxicity of CIK cells against human lung adenocarcinoma by upregulating MICA and IL-2 secretion

Xiao-yan Fan[1,*], Peng-yu Wang[2,3*], Chao Zhang[3], Yu-long Zhang[4], Yun Fu[3], Cong Zhang[2], Qiao-xia Li[2], Jie-na Zhou[2] , Bao-en Shan[3] & Dong-wei He[2]

[1]Department of Oncology, Hebei General Hospital, Shijiazhuang, Hebei, 050000, People's Republic of China. [2]Department of Clinical Bio-Cell, 4th Hospital, Hebei Medical University, Shijiazhuang, Hebei, 050000, People's Republic of China. [3]Research Center, 4th Hospital, Hebei Medical University, Shijiazhuang, Hebei, 050000, People's Republic of China.[4]Department of Surgery, Number One Hospital of Shijiazhuang, Shijiazhuang, Hebei, 050000, People's Republic of China. [*]These authors contributed equally to this work. Correspondence and requests for materials should be addressed to D.-W.H. (e-mail: chndongwhe@hotmail.com)

Supplementary Figure 1. Western blot assays were used to determine the changes of protein levels. GAPDH was utilized for an endogenous reference to standardize protein expression levels.

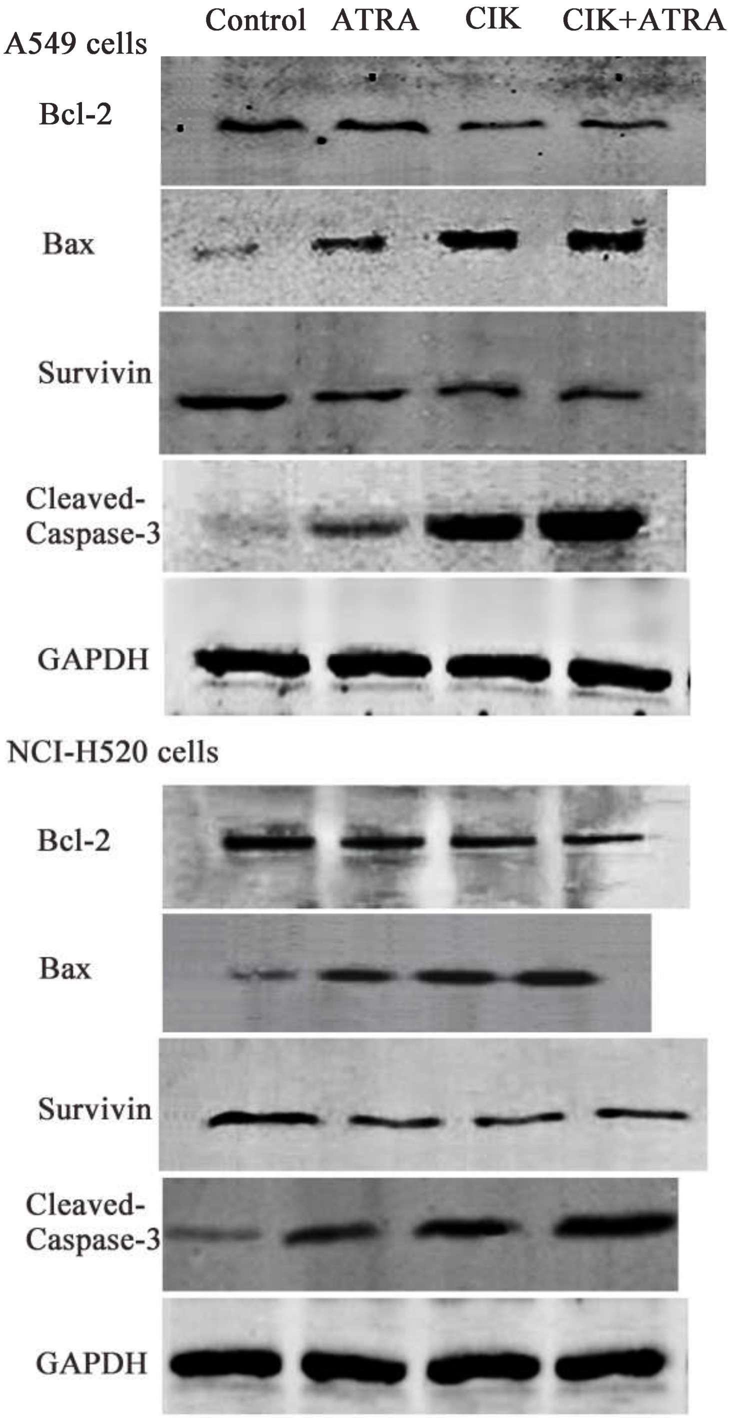
Control
ATRA
CIK
CIK+ATRA
A549 cells
Bcl-2
Bax
Survivin
Cleaved-Caspase-3
GAPDH
NCI-H520 cells
Bcl-2
Bax
Survivin
Cleaved-Caspase-3
GAPDH
